# 19p13.3 Deletion With Polyotia: A Case Report and Literature Review

**DOI:** 10.7759/cureus.19661

**Published:** 2021-11-17

**Authors:** Carlos Silvera Redondo, Camilo Andrés Avendaño Capriles, David Fernández Sánchez, Ricardo David Espinosa, Ana Sofía Acostamadiedo Marx

**Affiliations:** 1 Department of Genetics, Hospital Universidad del Norte, Barranquilla, COL; 2 Foundations of Clinical Research (FCR) Program, Harvard Medical School, Boston, USA; 3 Medicine, Universidad del Norte, Barranquilla, COL

**Keywords:** learning disabilities, congenital abnormalities, chromosome aberrations, chromosome deletion, microarray analysis

## Abstract

Mutations at chromosome 19 are rare, and reports in the literature are scarce and clinically variable. This chromosome has a high genetic density, and hence a given deletion can cause distinctive effects on body systems and, in addition, result in a characteristic phenotype.

We report the case of a patient who presented with distinctive signs and symptoms such as delayed psychomotor development, severe postnatal delay, dolichocephaly, polyotia, and ocular hypertelorism. Even though all cases with a chromosome 19 deletion do not present in the same way, they still share some clinical manifestations that should be considered, which prompted us to present a summary of the available literature on the subject. Additionally, to our knowledge, this is the first and only case with polyotia in its phenotype to be reported in Colombia to date.

## Introduction

During the last few years, several new syndromes have been described in the field of clinical genetics thanks to the development and increasing use of tests such as comparative genomic hybridization (CGH) arrays. However, the information available regarding 19p deletions is still scant [1]. As of 2021, only 85 cases have been reported in the DECIPHER database [2]. The 19p13.3 deletion syndrome is usually sporadic, meaning it appears de novo, resulting from a chromosomal segment deletion during the formation of reproductive cells or in early fetal development. The majority of the affected individuals have no history of the disease in their families [3]. To our knowledge, only seven patients had documented parental origin [2].

The 19p13.3 region contains 6.9 Mb and several different genes within. Hence, depending on the number of bases compromised by a deletion, this will result in a specific clinical presentation. For example, this chromosomal region contains the serine/threonine kinase 11 (STK11) gene, associated with Peutz-Jeghers syndrome, which codes for a serine-threonine kinase protein that acts as a cell cycle metabolism regulator, cell polarity modulator, and tumor suppressor [3].

Additionally, other genes within this region are frequently affected by these deletions, such as the testicular haploid expressed gene (THEG), which plays an essential role in body development through its influence on hormone development, and whose absence may impact the height of the patients, who are generally short in stature [1]. The product of SHC adaptor protein 2 (SHC2) is responsible for the maturation of sensory, cortical, and sympathetic neurons [4], and hence its loss leads to impaired vision and hearing [1]. The actin alpha 1 (ACTA1) gene is expressed in neuronal tissue, especially in the cerebellum and hippocampus, where mutations in this gene have been associated with hypotonia and delayed motor skills development [5]. G protein subunit alpha 11 (GNA11) is related to normal craniofacial and cardiovascular development, and transducin-like enhancer protein 2 (TLE2), which is associated with neurogenesis and epithelial differentiation during embryonic development [6]. In addition, deletions at this level have been associated with different pathologies related to deficits in tumor suppression, such as breast cancer and lymphomas [7,8]. Given the broad clinical spectrum, the prognosis and life expectancy will depend on the manifestations in each patient.

In the silico genomic analysis of 19p13.3 microdeletion, breakpoints revealed numerous highly repetitive sequences, suggesting events mediated by long interspersed nuclear elements (LINEs) and/or short interspersed nuclear elements (SINEs) as the generators of these microdeletions [1]. These genetic alterations give rise to the phenotypic spectrum of the disease, which is still being defined. In this report, we explore the clinical features of a patient who presented for an appointment at the Hospital of the Universidad del Norte, and to the best of our knowledge, this is the first reported case of this clinical syndrome in Latin America.

## Case presentation

A seven-year-old male patient presented to the clinical genetics office accompanied by his mother. She was concerned because her child had experienced learning difficulties and growth retardation since his first year of life. The patient had no relevant comorbidities, and his parents were of appropriate age and height, not consanguineous, and had four children (one deceased, who had suffered from Down syndrome). No prenatal history was available.

Physical examination revealed delayed language and fine motor skills, as well as low height (112 cm; Z-score: -1,87; percentile: 3%) and weight (17 kg; Z-score: -1.61; percentile: 5%). There was a prominent sagittal suture with dolichocephaly, skull bulging, triangular facies, short and oblique fissures, epicanthus, telecanthus, ocular hypertelorism, low bridge, hypoplasia of the distal third of the eyebrows, and low-set, winged, and rotated pinnae, with bilateral polyotia (perceived as soft preauricular skin-covered nodules). No stereotypies (repetitive movements or sounds) were observed. No pictures of our patient's phenotypical findings are shown here, as his mother did not give permission to publish them.

At the time, the patient's mother brought an old report of her son's bone age, which was found to be of 30 months, for a chronological age of five years. Additionally, she brought a karyotype test, with a normal result. Due to his learning disabilities, fragile X testing was requested. The fragile X study reported the presence of an allele of 28 CGG repeats in the analyzed region of FMR1, which was a normal result. Finally, a microarray analysis was ordered to rule out other possibilities.

**Figure 1:**
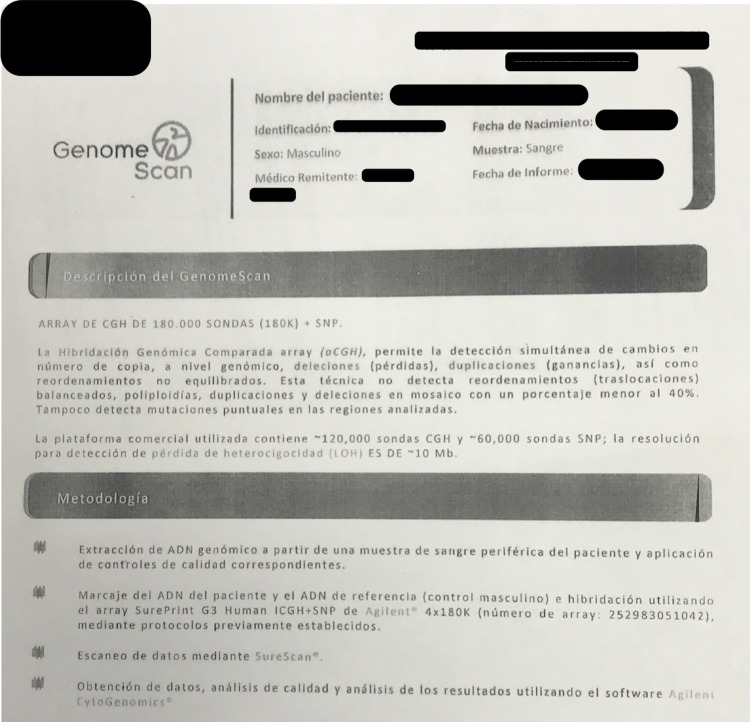
Microarray report in Spanish (description and methodology) Translation: Genome scan description: - Comparative genome hybridization array (ACGH) allows simultaneous detection of changes in the number of copies, on a genomic level, deletions (losses), duplications (increases), as well as unbalanced rearrangements. This technique does not detect balanced rearrangements (translocations), polyploidies, mosaic duplications, and deletions with a percentage below 40%. Neither does it detect punctual mutations in the analyzed regions - The commercial platform used contains approximately 120,000 CGH probes and 60,000 SNP probes; the resolution for the detection of loss of heterozygosity (LOH) is approximately 10 Mb Methodology: - Extraction of genomic DNA from a peripheric blood sample from the patient and the application of the corresponding quality checks - Marking of the patient’s DNA and reference DNA (masculine control) and hybridization using the array Sureprint G3 Human ICGH+SNP from Agilent 4x180k (array number: 252983051042), through previously established protocols - Data scanning through Surescan - Data obtention, quality analysis, and analysis of results using Agilent software Cytogenomics

**Figure 2:**
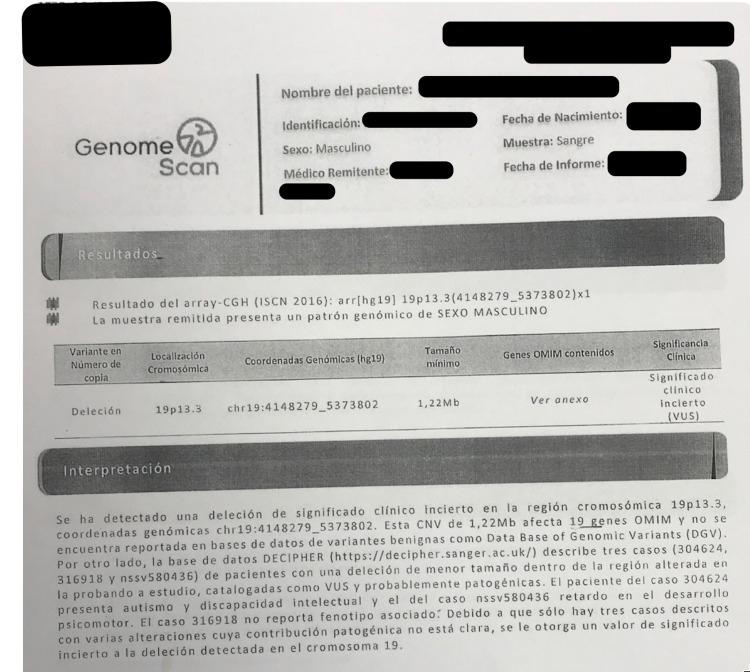
Microarray report in Spanish (results and interpretation) Translation: Results: - Result of the array-CGH (ISCN 2016): arr[hg19] 19p13.3(4148279_5373802)x1 - The remitted sample presents a genomic pattern of masculine sex Interpretation: - An uncertain clinical significance deletion has been detected on the chromosomic region 19p13.3, genomic coordinates chr 19:4148279_5373802. This copy number variant of 1.22 Mb affects 19 OMIM genes and is not reported in databases of benign variants such as the Data Base of Genomic Variants (DGV). On the other hand, the DECIPHER database (https://decipher.sanger.ac.uk/) describes three cases (304624, 316918, and nssv580436) of patients with smaller deletions inside the altered region in the proband of study, cataloged as VUS and probably pathogenic. The patient of case 304624 presents autism and intellectual disability and the case nssv580436 presents psychomotor delay. The case 316918 does not report an associated phenotype. Because there are only three described cases with several alterations whose pathogenic contribution is not clear, it is granted an uncertain clinical significance to the deletion detected in chromosome 19

The microarray report (Table 1) showed a 19p13.3 deletion of 1.22 Mb that involved 19 genes and stated that it was of uncertain significance given that only three similar cases had been described so far, all with smaller deletions (Figures 1, 2 in the Appendix). One case presented autism and intellectual disability, another had delayed psychomotor development, and the third one did not have an associated phenotype, according to the report.

**Table 1:** Microarray results

Variant in copy number	Chromosomic localization	Genomic coordinates (hg19)	Minimum size	OMIM genes contained	Clinical significance
Deletion	19p13.3	Chr19:4148279_5373802	1,22 Mb	See full report in Appendix (Figures 1, 2)	Variant of uncertain significance (VUS)

Due to his growth abnormalities, the patient was referred to endocrinology. Also, physical and occupational therapy every three weeks was selected as a therapeutic approach, and an abdominal ultrasound was ordered. However, there are no follow-up reports available because the patient stopped attending appointments.

## Discussion

The information currently available on the 19p13.3 deletion syndrome is limited, and the reports mention variable clinical features. For example, de Smith et al. have described a patient with overgrowth, macrocephaly, obesity, mental retardation, and behavioral problems (self-and hetero-aggressions and temper tantrums) [9]. Sibberg et al. have described two patients, both with aberrations in chromosome 19. However, they had different manifestations. The first case was a two-year-old patient with macrocephaly, normal growth, and a 1.25 Mb deletion at the 19p13.3 site. The other was a nine-year-old patient with microcephaly, growth retardation, and duplication of 0.81 Mb at the same location. Nevertheless, both patients had dysmorphic features and delayed psychomotor development [10].

Another publication has reported hypotonia, congenital cardiac malformations, sensorineural and conductive hypoacusis, absence seizures, tonic-clonic seizures, difficulty in social development, short philtrum, thick eyebrows, keloids, immune dysregulation, dysmorphic features, and again, delayed psychomotor development, but with normal postnatal growth [11].

Lastly, Peddibhotla et al. have discussed a cohort of eight patients with 19p13.3 microdeletion, a high incidence of learning difficulties, hypotonia, and global developmental delay, along with the considerable presence of congenital anomalies such as feeding difficulties, congenital heart disease, and gastrointestinal, renal, urogenital, auditory, and visual anomalies. Other abnormalities found in the patients were ventriculomegaly, broad forehead, mid-facial hypoplasia, low-set ears, smooth philtrum, sunken eyes, and VACTERL association [1].

The clinical presentation of our patient was compatible with what has been presented in the literature, which is summarized in Table 2 [1,3,6,9-17]. Particularly, he showed learning difficulties (language delay) and postural and motor abnormalities (delayed fine motor skills). However, our literature search did not yield any reports of the polyotia that our patient presented, since, to our knowledge, this is the first reported case of polyotia related to a 19p13.3 deletion.

**Table 2:** Clinical characteristics found in different studies Yes: present in all patients; No: not present in any patient; -: not reported in any patient; ( ): used when only some patients out of the total had the finding

General symptoms	Specific symptoms	Peddibhotla et al., 2013 [1]	Souza et al., 2011 [3]	Al-Kateb et al., 2010 [6]	de Smith et al., 2011 [9]	Siggberg et al., 2010 [10]	Archer et al., 2005 [11]	Sgardioli et al., 2018 [12]	Campoverde et al., 2016 [13]	Nevado et al., 2015 [14]	Kuroda et al., 2014 [15]	Risheg et al., 2013 [16]	Scollon et al., 2013 [17]
Neurological/neurodevelopmental disorders	Intellectual disability	-	-	Yes	Yes	Yes	Yes	Yes	-	Yes	Yes (1/6)	Yes	No
	Developmental delay	Yes (7/8)	Yes	Yes	Yes	Yes	Yes	Yes	Yes	Yes	-	Yes	Yes
	Speech delay	Yes (4/8)	Yes	Yes	Yes	Yes	No	Yes	-	Yes (8/11)	-	Yes	Yes
	Motor delay	Yes (1/8)	Yes	Yes	No	Yes	Yes	Yes	-	-	-	Yes (1/3)	Yes
	Learning disabilities	Yes (7/8)	Yes	Yes	Yes	Yes	Yes	Yes	-	-	-	No	Yes
	Memory impairment	No	No	-	No	-	-	No	-	-	-	No	No
	Seizures	Yes (1/8)	Yes	-	No	-	Yes	-	Yes	-	Yes (4/6)	No	Yes
	Hypotonia	Yes (6/8)	Yes	Yes	No	Yes (1/2)	Yes	Yes	Yes	-	-	Yes	No
	Unsteady gait	No	No	Yes	No	No	-	Yes	-	Yes	-	Yes (1/3)	No
Psychiatric disorders	Attention deficiency	No	No	-	No	-	-	No	-	-	-	Yes (1/3)	No
	Behavioral problems	No	No	-	Yes	-	-	Yes	-	-	-	No	No
	Anxiety	Yes (1/8)	No	-	No	-	-	Yes	-	-	-	No	No
Craniofacial abnormalities	Macrocephaly	No	No	Yes	Yes	Yes	No	Yes	-	Yes	-	Yes (1/3)	No
	Facial dysmorphisms	Yes	Yes	Yes	Yes	Yes	Yes	Yes	-	Yes	-	Yes	No
	High and/or wide forehead	Yes (5/8)	No	Yes	Yes	Yes (1/2)	Yes	Yes	-	Yes	Yes	Yes (2/3)	No
	Otic malformations	Yes (6/8)	No	Yes	No	Yes (1/2)	Yes	Yes	-	Yes	Yes	Yes	No
	Deafness	Yes (4/8)	No	Yes	No	No	Yes	-	-	Yes	Yes	Yes (1/3)	No
	Hair implantation anomalies	No	No	Yes	Yes	-	Yes	Yes	-	Yes	-	Yes (2/3)	No
	Wide eyebrows	-	No	-	No	-	Yes	-	-	Yes	Yes	Yes (1/3)	No
	Long face	Yes (1/8)	No	-	No	Yes (1/2)	Yes	-	-	Yes	Yes	Yes (1/3)	No
	Pointed chin	Yes (1/8)	No	-	No	-	-	-	-	Yes	Yes	No	Yes
	Thin lips	No	No	-	No	-	Yes	-	-	Yes	Yes	No	No
	Smooth philtrum	Yes (4/8)	No	-	No	-	Yes	-	-	Yes	Yes	Yes (2/3)	No
	Low hanging columella (nasal columella)	No	No	-	No	Yes (1/2)	-	-	-	Yes	Yes	No	No
	Epicanthal folds	Yes	Yes	Yes	No	-	-	Yes	-	Yes	-	No	No
	Nasal dysmorphisms	Yes (2/8)	Yes	Yes	Yes	Yes (1/2)	Yes	Yes	-	Yes	-	No	No
	Mouth dysmorphisms	No	No	Yes	Yes	-	No	Yes	-	Yes	-	Yes	No
	Palatal abnormalities/velopharyngeal insufficiency	Yes (3/8)	No	Yes	No	-	Yes	No	-	Yes	Yes	Yes (2/3)	Yes
Gastrointestinal abnormalities	Gastroesophageal reflux	Yes (2/8)	No	Yes	No	-	-	Yes	Yes	Yes	-	Yes (1/3)	No
	Dysphagia	Yes (1/8)	No	-	No	-	-	Yes	Yes	Yes	-	No	No
	Hyperbilirubinemia at birth	No	No	-	No	Yes (1/2)	-	Yes	-	Yes	-	No	No
Immunological alterations	Recurrent sinopulmonary infections	No	No	-	No	Yes (1/2)	Yes	-	Yes	Yes	-	No	No
	Hypo-IgG	No	No	-	No	-	Yes	-	Yes	Yes	-	No	No
	Immunodeficiency	No	No	-	No	-	Yes	-	Yes	Yes	-	No	No
Ocular abnormalities	High myopia	Yes (1/8)	Yes	-	No	Yes (1/2)	-	-	-	Yes	Yes	Yes (1/3)	No
Limb abnormalities	Joint and extremity edema	No	No	-	No	-	-	Yes	-	-	-	Yes (1/3)	No
	Finger and toe abnormalities	Yes (1/8)	Yes	Yes	Yes	-	Yes	Yes	Yes	-	-	Yes	Yes
Bone abnormalities		No	Yes		No	No		Yes	-	-	-	Yes (1/3)	
Endocrinopathies	Growth retardation	Yes (6/8)	No	Yes	No	Yes	No	Yes	Yes	-	Yes	No	No
CNS malformations	Hypoplasia of the corpus callosum	No	No	-	No	-	-	-	Yes	-	-	Yes (1/3)	No
	Ventriculomegaly	Yes (2/8)	No	-	No	-	-	-	Yes	-	-	No	No
Congenital heart disease		Yes (6/8)	Yes	-	No	-	-	-	-	-	Yes	Yes	No
Prenatal	Normal karyotype	-	Yes	Yes	Yes	Yes	-	-	Yes	-		Yes	Yes
	Intrauterine growth restriction	Yes (4/8)	No	No	No	Yes (1/2)	Yes	-	Yes	-	Yes	-	No
Neonatal	Low birth weight	Yes (4/8)	No	No	No	Yes (1/2)	Yes	-	Yes	-	Yes	-	-
	Dysmorphic features	Yes	Yes	Yes	Yes	Yes	Yes	-	Yes	-		Yes	No
Others	NF-1	No	No	-	No	-	-	-	-	Yes	Yes (1/6)	No	No
	Hernias	Yes (2/8)	No	-	No	-	Yes	-	-	Yes	Yes (1/6)	No	No
	Peutz-Jeghers phenotype (PJS)	No	Yes	-	No	-	-	-	-	Yes	Yes	No	Yes
	Hemihyperplasia	Yes (1/8)	No	-	No	-	-	-	-	Yes	Yes (1/6)	No	No
	Aplasia cutis	No	-	Yes	-	Yes	No	-	-	-	-	Yes	-

Unfortunately, the patient's attendance during the follow-up period was poor, which limited his clinical follow-up. Therefore, some clinical features of this syndrome that were not detected may still be present in our patient.

## Conclusions

Despite the paucity of literature available on this disease, the case presented still provides good insight into some of the manifestations found in these patients. Additionally, we found other clinical characteristics that may be present if a clinician encounters a patient with this challenging diagnosis, highlighting the importance of keeping this disorder in mind for a differential diagnosis in patients with dysmorphic features and developmental delay.
